# Can omentin-1 be a prognostic marker in surgical intensive care patients?

**DOI:** 10.3906/sag-2009-158

**Published:** 2021-10-21

**Authors:** Yücel GÜLTEKİN, İsmail BİRİ, Afig GOJAYEV, Selen YILMAZ IŞIKHAN, Oytun PORTAKAL AKÇİN, Yusuf Alper KILIÇ

**Affiliations:** 1 Department of General Surgery, Faculty of Medicine, Uşak University, Uşak Turkey; 2 Deparment of General Surgery, Koru Ankara Hospital, Ankara Turkey; 3 Department of Surgical Oncology, Faculty of Medicine, Ankara University, Ankara Turkey; 4 Deparment of Biostatistics, Vocational Higher School of Social Sciences and Faculty of Medicine, Hacettepe University, Ankara Turkey; 5 Deparment of Medical Biochemistry, Faculty of Medicine, Hacettepe University, Ankara Turkey; 6 Deparment of General Surgery, Faculty of Medicine, Hacettepe University, Ankara Turkey

**Keywords:** Intensive care, omentin, prognostic marker

## Abstract

**Background/aim:**

A member of the adipokine family, omentin-1 is selectively secreted from visceral fat tissue and the omentum. It has been shown that omentin-1 is involved in the pathogenesis of certain diseases and can be used as a prognostic marker. This study first investigated the prognostic significance of omentin-1 in surgical intensive care patients. In addition, the relationship between omentin-1 and laboratory and clinical parameters commonly used in intensive care units (ICUs) was evaluated.

**Materials and methods:**

One hundred and fifty-four patients hospitalized in the surgical ICU were included in the study. Blood samples for omentin-1 were collected from the patients displaying clinical condition changes. Changes in omentin-1 levels were observed during the hospital stay of the patients. A total of 423 blood samples were evaluated. Omentin-1 levels were compared to the laboratory parameters routinely monitored in the ICU and the prognostic significance of omentin-1 for surgical intensive care patients was investigated.

**Results:**

The median APACHE II score of all patients was (median-IQR, 8.0–6.0 ng/mL). Omentin-1 levels of the alive patients in the ICU (median-IQR, 339.04–407.68 ng/mL) were significantly higher compared to dead patients (median-IQR, 166.40–363.60 ng/mL). Omentin-1 levels were higher in nonsepsis patients compared to the levels of the patients in sepsis and septic shock (p < 0.001). Omentin-1 values were negatively correlated with the C-reactive protein and procalcitonin levels, body temperature, and the SOFA (sequential organ failure assessment score) scores and they were positively correlated with albumin, prealbumin, and glucose levels.

**Conclusion:**

Omentin-1 may play a role in the complex constructs of inflammation and metabolic events in intensive care patients. Reduced omentin-1 levels in surgical intensive care patients were associated with poor prognosis and increased mortality.

## 1. Introduction

Adipose tissue is an endocrine organ, from which various mediators called adipokines are secreted. Many adipokines secreted from the adipose tissue have been identified including adiponectin, visfatin, resistin, and leptin [1,2].

Omentin, which is a member of the adipokine family and also known as intellectin, was first isolated from intestinal Paneth cells in 2005 [2,3]. It acts as a secondary messenger in some important cellular events such as glucose metabolism, cell proliferation, and apoptosis. In vitro studies on cells isolated from human adipose tissue have found out that omentin stimulates insulin signal transduction and enhances glucose transport by activating protein kinase Akt/protein kinase B [3]. Studies have reported that omentin-1 is closely related to inflammation and plays a role in the pathogenesis of inflammation. Omentin-1 is a prognostic marker in some diseases, such as coronary artery disease and cerebrovascular disease [4,5].

Omentin-1 is selectively expressed more in the omentum and visceral adipose tissue compared to other members of the adipokine family [2,6]. In the light of this thought, the feasibility of the use of omentin-1 as a biomarker for surgical intensive care patients was evaluated in this study. Furthermore, the relationship between omentin-1 and laboratory parameters commonly used in intensive care units (ICUs) was investigated.

## 2. Materials and methods

### 2.1. Study design

To test omentin-1 levels, 423 blood samples were collected from inpatients admitted to our tertiary-care surgical ICU in the period from June 2017 to December 2017. Patients aged less than 18 years, those who stayed in the ICU for 24 h or less and pregnant patients were excluded from the study. The study cohort consisted of 154 patients with 82 males and 72 females. The study protocol was approved by the local ethics committee (Hacettepe University Ethics Committee, GO 16/490-56) and the study was conducted in compliance with the ethical standards stated in the Declaration of Helsinki. Written informed consent was obtained from the patient or the patient’s spouse or legal guardian. Patient data and samples were collected prospectively. The presence of septic disease was defined according to the Third International Consensus Definitions for Sepsis (Sepsis-3) [7]. Patients with documented or suspected infection and life-threatening organ failure were accepted as sepsis. A 2-point increase in SOFA score was used to define organ dysfunction. The patients who had serum lactate value >2 mmol/L and who received vasopressor therapy to ensure that the arterial pressure is 65 mmHg despite sufficient fluid resuscitation were evaluated as septic shock. Crystalloids were primarily preferred in fluid therapy in sepsis patients, norepinephrine was administered primarily in patients in need of vasopressors, and dopamine was preferred as a secondary agent in these patients. The primary purpose of the study was to search for an answer to the question of whether omentin-1 can be a prognostic marker in surgical intensive care patients or not. The relationship of omentin-1 with inflammatory markers such as C-reactive protein (CRP) and procalcitonin (PCT) was investigated as a secondary purpose. In addition, omentin-1 level was evaluated with other laboratory parameters and scoring systems used in the monitoring of intensive care patients.

### 2.2. Collection and analysis of blood samples

Baseline blood samples were collected at the time of admission of the patients to the ICU. Omentin-1 was evaluated simultaneously with the alterations in the vital signs and clinical care of the patients (in case of an increase of two points in the SOFA score; 24 h later after the patient started or stopped oral intake, and in patients, who developed sepsis and septic shock). Blood samples for omentin-1 were again collected from the patients at the time of discharge from ICU. Routine laboratory tests, vital signs, and the nutritional status of the patients were evaluated daily and the results were recorded. Blood samples were centrifuged and the serum samples were kept at –80 ºC until the time of the analysis.

Serum omentin-1 was measured by sandwich based ELISA (BioVendor, GmbH, Germany). Standards, 2–64 ng/mL, were prepared, and serum samples were diluted. Total 100 µL of samples were incubated in microtiter wells coated with polyclonal antihuman omentin-1 antibody for capturing omentin in the sample. After washing, biotin-labelled polyclonal antihuman omentin antibody was added and incubated. Streptavidin-HRP conjugate was added following washing step, and incubated. After another wash, the substrate solution (TMB) was added, the reaction was stopped by an acidic solution and absorbance of the product is measured. The absorbance was directly proportional to the concentration of omentin in the sample. The concentration of each sample was determined from the standard curve provided in the same assay. Each sample result was expressed as ng/mL. Limit of detection of the measurement was 0.5 ng/mL. Intra-assay precision and inter-assay precision were, 4.1 ng/mL and 3.7 ng/mL, and 4.8 ng/mL and 4.4 ng/mL at low and high concentrations, respectively.

### 2.3. Statistical analysis

Descriptive statistics of numerical variables are summarized as mean ± standard deviation or median-interquartile ranges (IQR) based on the data distribution in the groups. Categorical variables were expressed as percentages. Comparisons of the mean values of the parameters between the survivors and nonsurvivors were performed by the independent-sample t-test when the data was normally distributed in the groups. The Mann–Whitney U test was used for comparing the groups with data not conforming to a normal distribution. A Chi-square test was used for comparing the percentages between the groups. Relationships between omentin-1 levels and other clinical parameters were evaluated with Spearman’s correlation test. Additionally, to identify the relationship between two or more variables, the package rmcorr was used for calculating the correlation based on repeated measurements from the same patient. To determine the cut-off level for omentin-1, receiver operating characteristics (ROC) curves were created by plotting sensitivity against 1-specificity. The prognostic values of the variables were tested by conducting univariate and multivariable Cox regression analyses. All variables with p ≤ 0.10 in the univariate cox analysis were included in the multiple cox regression. All statistical analyses were performed by using IBM SPSS Statistics v: 22.0 and R statistical computing language v: 3.4.3. The statistical significance level was accepted at a p-value of < 0.05.

## 3. Results

A total of 423 omentin-1 measurements were performed in the study. The change in the levels of omentin-1 was evaluated in patients during their stay at ICU. The characteristic features of patients, who died or survived in ICU, are summarized in Table 1. 

**Table 1:** Main clinical characteristics of the patients based on the last status.

Parameter	Survivor (n = 135)	Dead (n = 19)	P value
Age (years)	18–61.50	25–63.50	0.993
Sex (male/female)	71/64	11/8	0.665
BMI (kg/m2)	27.08 ± 3.78	25.45 ± 4.46	0.173
Charlson comorbidity index	5–3	6–3	0.051
Operated/not operated	96/39	11/8	0.312
APACHE II score	5–10	8–13	0.004
SOFA score	3–5	6–13	< 0.001
Blood glucose (mg/dL)	160.10 ± 53.32	131.38 ± 38.22	< 0.001
Body_temprature (°C)	36.6 ± 0.6	39.7 ± 29.5	0.231
Total protein (g/dL)	5.75 ± 0.73	5.65 ± 0.97	0.286
Albumin (g/dL)	2.85 ± 0.48	2.59 ± 0.44	< 0.001
Creatinine (mg/dL)	1.08 ± 0.94	2.04 ± 1.46	< 0.001
Hemoglobin (g/dL)	11.01 ± 1.77	11.31 ± 12.23	0.784
Hematocrit (%)	32.90 ± 5.90	28.98±4.86	< 0.001
Prealbumin (mg/dL)	14.13 ± 7.11	11.86 ± 4.88	0.028
Procalcitonin (ng/mL)	5.25 ± 12.00	18.74 ± 27.24	< 0.001
CRP (mg/L)	17.63 ± 11.50	20.48 ± 11.48	0.107
WBC(103/µL)	11.06 ± 5.55	12.20 ± 5.94	0.063
RDW (%)	16.3 ± 3.57	17.17 ± 3.07	0.034
Monosit (103/µL)	0.50–0.70	0.50–0.55	0.199
Platelet count	158–211.5	109–163	< 0.001
Omentin-1 (ng/mL)	339.04–407.68	166.40 - 363.60	< 0.001

Mean ± std. deviation, median-IQR (interquartile range) BMI; Body mass index, APACHE II; Acute physiology and chronic health evaluation, SOFA; Sequential organ failure assessment score, CRP; C-reactive protein, WBC; White blood cell, RDW; Redcell distribution width.

**Figure 1:**
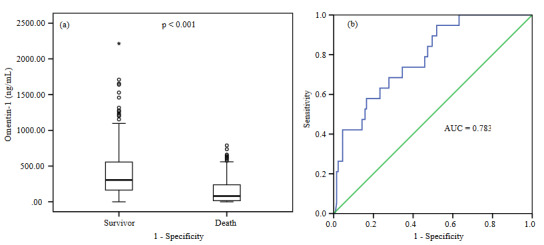
(a) Omentin-1 levels of patients, who died in the ICU were lower than omentin-1 levels of patients, who survived in the ICU. (b) The cut-off value of the serum omentin-1 level for survivors and nonsurvivors is 238.42 ng/mL (AUC = 0.783, p < 0.001). The cut-off value corresponds to the sensitivity and the specificity values of 0.74 and 0.65, respectively (PPV = 52%, NPV = 91%).

**Figure 2:**
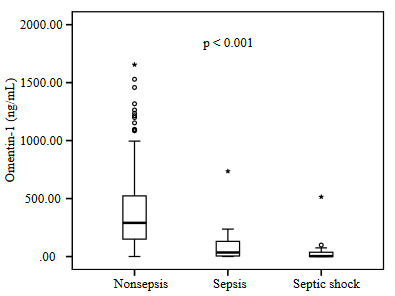
A total of 423 evaluations were made for omentin-1 levels. While sepsis and septic shock criteria were met by 42 and 32 patients, respectively; omentin-1 levels were higher (median-IQR, 290.80–374.32 ng/mL) in nonsepsis patients compared to the levels found in sepsis patients (median-IQR, 35.22–131.70 ng/mL, p < 0.001) and patients with septic shock (median-IQR, 4.00–54.62 ng/mL, p < 0.001).

Omentin-1 levels were significantly higher in survivors compared to nonsurvivors (Figure 1a). No significant difference was found between the patients who underwent operation (310.0–357.3 ng/mL) and those who did not (333.5–471.0 ng/mL) in terms of the median omentin-1 level (p = 0.80). The predictive value of omentin-1 in estimating mortality in ICU was analyzed with the ROC curve (Figure 1b). The cut-off value of omentin-1 for mortality prediction was 238.42 ng/mL. Patients with an omentin-1 value less than or equal to this cut-off value were classified as critical whereas patients with an omentin-1 value higher than this value were considered likely to survive. A subgroup analysis for sepsis was performed on the patients. Omentin-1 values were higher in nonsepsis patients compared to the levels of the patients with sepsis and septic shock (Figure 2).

**Figure 3:**
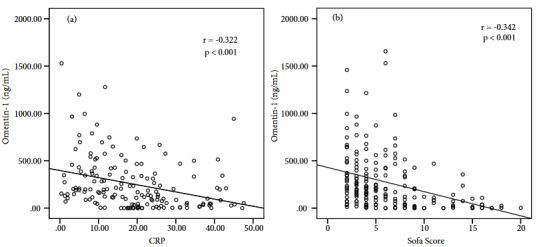
(a) A negative correlation was found between the levels of omentin-1 and CRP (r = –0.322, p˂ 0.001). (b) There was a negative correlation between omentin-1 levels and the SOFA scores (r = –0.342, p < 0.001).

Correlation analyses were performed between the omentin-1 levels and routine parameters used in ICU (Table 2). A negative and significant correlation of omentin-1 levels was found with CRP (Figure 3a) and PCT levels, body temperature, and the SOFA scores (Figure 3b). Albumin and blood glucose levels were statistically significantly and positively correlated with the omentin-1 levels. A negative and insignificant correlation was found between the APACHE II scores and omentin-1 levels.

**Table 2:** Correlations between omentin-1 and other parameters.

	Omentin (n = 423)	
	No adjustment	Adjusted for dependency
SOFA score	–0.342**	–0.244**
APACHE II score	–0.139	–0.058
Blood glucose	0.209**	0.140*
Creatinin	–0.267**	–0.029
Hematocrit	0.145**	0.086
Haemoglobin	0.069	0.004
WBC	–0.099	–0.132*
Procalcitonin	–0.189*	–0.134*
CRP	–0.322**	–0.333**
RDW	–0.002	0.002
Platelet	0.215**	0.036
BMI	–0.043	–0.028
Body temperature	–0.233**	–0.161**
T. protein	0.071	0.076
Albumin	0.303**	0.252**
Prealbumin	0.161	0.136*

**Correlation is signiﬁcant at the 0.01 level. *Correlation is signiﬁcant at the 0.05 level. SOFA; Sequential organ failure assessment score, APACHE II; Acute physiology and chronic health evaluation, WBC; White blood cell, CRP; C-Reactive rrotein, RDW; Redcell distribution width, BMI; Body mass index.

The ICU patients constituted three groups as follows: enterally-fed patients, TPN (total parenteral nutrition) receiving patients, and the patients, who did not undergo any of these two modes of feeding but received only parenteral fluids. There was a significant difference in the distribution of omentin-1 levels in all three groups (p < 0.001). The omentin-1 level (median-IQR, 80–199.50 ng/mL) of the TPN group was statistically significantly lower compared to the level of the parenteral fluid-only group (median-IQR 240–360 ng/mL) and the level of the enterally-fed group (median-IQR, 304.96–389.92 ng/mL) (p < 0.001 and p ˂ 0.001, respectively). However, there was not a statistically significant difference between the parenteral fluid-only group and the enterally-fed group (p = 1.000).

Variables (t-tests) affecting the final patient outcomes (survival or death) in ICU were included in the univariate Cox model. Previous studies have shown that an APACHE II score of 10 or more is associated with a poor prognosis [8]. APACHE II scores >10 and omentin-1 levels were included in the model as variables based on the cut-off values. According to the univariate analysis; omentin-1, the SOFA scores and APACHE II scores and hematocrit were found to be effective factors on survival. As a result of the multivariable analysis; the SOFA score, the levels of creatinine, and omentin-1 were identified as independent significant parameters in predicting the survival in ICU (Table 3).

**Table 3:** Univariate and multivariable Cox regression analysis for omentin-1 and other clinical factors to predict survival at the ICU.

	Univariate analysis		Multivariable analysis	
Parameter	UnadjustedHR (95% CI)	p- value	AdjustedHR (95% CI)	p-value
SOFA score	1.48 (1.15–1.90)	0.002	1.138 (1.006–1.288)	0.040
APACHE II score (>10)	3.93 (1.23–12.53)	0.021	3.864 (0.693–21.550)	0.123
Charlson comorbidity index	1.234 (0.992–1.535)	0.059	0.915 (0.720–1.162)	0.467
Blood glucose	0.995 (0.981–1.009)	0.485	-	-
Albumin	0.301 (0.085–1.057)	0.061	0.483 (0.207–1.124)	0.091
Creatinine	1.263 (0.965–1.651)	0.089	0.513 (0.307–0.857)	0.011
Hematocrit	0.877 (0.792–0.971)	0.011	1.039 (0.990–1.089)	0.122
Procalcitonin	1.032 (0.997–1.067)	0.070	1.013 (0.999–1.028)	0.074
RDW	1.039 (0.907–1.191)	0.577	-	-
Omentin-1 (≤238.42 ng/mL)	1.102 (1.011–1.200)	0.027	2.274 (1.094–4.726)	0.028
Platelet	0.996 (0.992–1.001)	0.111	-	-

SOFA; Sequential organ failure assessment score, APACHE II; Acute physiology and chronic health evaluation, RDW; Redcell distribution width.

## 4. Discussion

Adipose tissue is considered an important component of metabolism and inflammation via the expression of various cytokines called adipokines [9,10]. This study has evaluated the change in omentin-1 levels at the time of admission and care of patients treated in the general surgery ICU. The role of omentin-1 in both prognosis and inflammation was analyzed.

Omentin is considered in the category of antiinflammatory adipokines [11,12]. Antiinflammation has been suggested to occur by c-Jun N-terminal kinase and cyclooxygenase-2 inhibition, possibly as a result of stimulation of pathways containing AMP-activated protein kinase and nitric oxide [13]. Urbanova et al. examined omentin-1 serum concentrations in patients with morbid obesity and type 2 diabetes mellitus and found a negative correlation between omentin-1 and CRP, which is a marker of acute inflammation [14]. Tan et al. evaluated omentin-1 in patients with polycystic ovaries and reported a negative correlation between omentin-1 and CRP [15]. These studies supported the antiinflammatory properties of omentin-1. However, Luedde et al. suggested that omentin-1 was not correlated with markers of infection and inflammation such as CRP, PCT, and interleukin-6 [16]. Results supporting the antiinflammatory characteristics of omentin-1 were obtained in this present study. A negative and significant correlation was found between omentin-1 and CRP and PCT.

Adipokines attract attention in sepsis studies because of their roles in inflammation and impaired glucose homeostasis [17,18]. The effects of adiponectin on metabolism and inflammation are similar to the effects of omentin from the adipokine family [19]. However, the predominant effect of leptin, another member of adipokines, is its pro-inflammatory effect [20]. Several studies about sepsis-adiponectin are available reporting both high and low levels of adiponectin in patients with sepsis. In addition, previous studies reported different results related to leptin in sepsis [21–24]. Luedde et al. reported in their study in medical intensive care that omentin-1 levels of patients with sepsis were not different compared to the levels of nonsepsis patients [16]. The diagnostic criteria of sepsis have been regularly updated; however, the opinion about the role of inflammation in sepsis has not yet changed. The omentin-1 levels of patients with nonsepsis were significantly higher compared to the levels of patients with sepsis in this present study (Figure 2). Many studies suggested the antiinflammatory properties of omentin-1. So, this was the outcome we expected. Luedde et al. evaluated omentin-1 level in the first three days of intensive care. However, in our study; the change of omentin-1 value was evaluated during the intensive care stay of the patients. We think that this situation is effective in obtaining a different result from Luedde et al. However, irregular and complex response to infection occurs in the host in sepsis. The hemostatic balance resulting from the complex interaction between proinflammatory and antiinflammatory responses is determinant for the patient’s condition [20]. Sepsis has a complex response to inflammation. In addition, patient populations in the studies are heterogeneous. Therefore, we think that contradictory results emerge in studies investigating adipokines in patients with sepsis. 

Both gene expression and plasma levels of omentin 1 in adipose tissue decrease in obese individuals. Omentin levels were lower compared to the normal population in obesity-related conditions such as type 2 diabetes mellitus and impaired glucose tolerance. Omentin is reported to be inversely correlated with BMI and fasting blood glucose [3]. Pan et al. also reported a negative correlation between omentin-1 and BMI and blood glucose levels in their study results [25]. However, Arjmand et al. reported that omentin levels were higher in cancer patients with BMI ≥ 25 and suggested that omentin may play a role in the development of obesity-related cancer in a systematic analysis evaluating omentin-1 levels, contrary to popular opinion [26]. A negative correlation was found between omentin-1 and BMI, but this was not significant in this study, contrary to expectation. There was also a positive correlation between blood glucose and omentin-1, contrary to what was expected. The complexity of intensive care patients and the regulatory role of omentin-1 in this complex structure and the heterogeneous patient population in the study may explain the emergence of this different result.

The common opinion is that enteral nutrition prevents intestinal barrier dysfunction, maintains mucosal integrity, and thus prevents bacterial translocation [27]. Significant improvements were observed in the intestinal barrier function only in half of the patients with acute pancreatitis fed with standard enteral nutrition in systematic analyses of the studies in the literature. Therefore, it has been reported that enteral nutrition acts favourably via some mechanisms of action other than the improvement in the intestinal barrier function [28]. Adipokines have been suggested to present a possible alternative mechanism for the therapeutic benefits of enteral nutrition [29]. Omentin-1 levels were found significantly higher in patients receiving enteral nutrition compared to TPN-receiving patients in our study. However, a difference was not found between the patient group receiving only parenteral fluids and the enterally-fed patient group. These results support the idea that adipokines play a role in ensuring intestinal mucosal integrity of enteral nutrition and preventing bacterial translocation. However, there is a need for further research on the subject. 

Scoring systems are frequently used in patient management in ICUs. The most widely used ones are the APACHE II and SOFA scoring systems. Luedde et al. reported no correlations between the APACHE II score and the omentin-1 levels [16]. An insignificant and weak correlation was observed between the APACHE II score and omentin-1 level in this present study. However, a significant and negative correlation was found between the SOFA score and omentin-1 level. The consecutive measurements of the SOFA scores are used for predicting mortality [30]. Our results suggest that the change in omentin-1 levels can be used for the prediction of prognosis in ICUs.

Adipokines can act as prognostic indicators in a variety of diseases via their direct effects on the regulation of hyperglycemia, glucose intolerance, and insulin resistance [20,23]. Wu et al. found a negative association between omentin-1 and cerebral infarction dimensions (r = –0.304, p ˂ 0.001). Patients with low omentin-1 serum levels (<129 ng/mL) had a higher risk of death than those with high serum omentin-1 levels (≥129.0 ng/mL) in the results of the study [4]. Narumi et al. reported in their study on patients with heart failure that patients with low omentin-1 level (IQR, 57–402 ng/mL) had a higher cardiac risk than high omentin-1 level (IQR, 323–661 ng/mL) [31]. The omentin-1 level (median-IQR, 166.40–363.60 ng/mL) was significantly lower in the dead patients compared to the omentin-1 level (median-IQR, 339.04-407.68 ng/mL) in the alive patients in this study. Low omentin-1 levels may be involved in poor prognosis and mortality as the effects of endothelial dysfunction, insulin resistance, abnormal glucose metabolism, and inflammation prevail [32]. It appears that the reduction in omentin-1 levels is consistent with poor prognosis and increased mortality in light of the above-mentioned data.

We are of the opinion that monitoring the trend in omentin-1 levels during the intensive care stay of patients is important in predicting mortality and prognosis instead of evaluating omentin-1 levels at the time of admission.

One of the strengths of this study is that patient groups are matched for age, weight, and BMI. Thus, the effect of confounding factors potentially to be involved in determining the effect of omentin-1 or other parameters have been mitigated, ensuring the reliability of results to identify variables acting on patient outcomes in intensive care. However, imbalances across the study groups cannot be completely excluded due to the complex clinical condition of intensive care patients. Due to small number of patients, the association between the serum omentin-1 level and individual comorbid disease could not be investigated. In addition, the groups included in the subgroup analyses resulted in low patient numbers across the study groups. These conditions constitute the limitations of our study. 

In conclusion, omentin-1 may play a role in the prognosis of intensive care patients with its effects on insulin resistance, abnormal glucose metabolism, and inflammation. This study showed that the omentin-1 value can be a prognostic marker for surgical intensive care patients and can be used to predict mortality. We think that studies on the subject will support the widespread use of omentin-1 as a prognostic indicator in surgical ICUs.

## Funding

This study was funded by Hacettepe University’s Scientific Research Coordination Unit.

## References

[ref1] Yang RZ Lee MJ Hu H Pray J Wu HB 2006 Identification of omentin as a novel depot specific adipokine in human adipose tissue: possible role in modulating insulin action American Journal of Physiology Endocrinology and Metabolism 290 1253 1261 10.1152/ajpendo.00572.2004 16531507

[ref2] Aktaş G Şit M Tekçe H 2013 New adipokines: leptin, adiponectin and omentin (in Turkish) Abant Medical Journal 2 56 62

[ref3] Batista CM Yang RZ Lee MJ Glynn NM Yu DZ 2007 Omentin plasma levels and gene expression are decreased in obesity Diabetes 56 1655 1661 17329619 10.2337/db06-1506

[ref4] Wu DM Wang S Wen X Han XR Wang YJ 2019 Impact of serum omentin-1 levels on functional prognosis in non-diabetic patients with ischemic stroke American Journal of Translational Research 11 1854 1863 30972209 PMC6456553

[ref5] Zhong X Zhang HY Tan H Zhou Y Liu F 2011 Association of serum omentin-1 levels with coronary artery disease Acta Pharmacologica Sinica 32 873 878 21602837 10.1038/aps.2011.26 PMC4003121

[ref6] Schaffler A Neumeier A Herfarth H Furst A Scholmerich J 2005 Genomic structure of human omentin, a new adipocytokine expressed in omental adipose tissue Biochimica et Biophysica Acta - Gene Structure and Expression 1732 96 102 10.1016/j.bbaexp.2005.11.005 16386808

[ref7] Singer M Deutschman CS Seymour CW Shankar-Hari M Annane D 2016 The third international consensus definitions for sepsis and septic shock (Sepsis-3) The Journal of the American Medical Association 315 801 810 26903338 10.1001/jama.2016.0287 PMC4968574

[ref8] Huang J Xuan D Li X Ma L Zhou Y 2017 The value of APACHE II in predicting mortality after paraquat poisoning in Chinese and Korean population: a systematic review and meta-analysis Medicine (Baltimore) 96 6838 6838 10.1097/MD.0000000000006838 PMC5627797 28746171

[ref9] Lau DCW Dhillon B Yan HY Szmitko PE Verma S. Adipokines 2005 : molecular links between obesity and atherosclerosis American Journal of Physiology-Heart and Circulatory Physiology 288 2031 2041 10.1152/ajpheart.01058.2004 15653761

[ref10] Senolt L Polanska M Filkova M Cerezo LA Pavelka K 2010 Vaspin and omentin: new adipokines differentially regulated at the site of inflammation in rheumatoid arthritis Annals of the Rheumatic Diseases 69 1410 1411 19914904 10.1136/ard.2009.119735

[ref11] Sengul E Duygulu G Dindar S 2013 Serum omentin-1, inﬂammation and carotid atherosclerosis in patients with non-diabetic chronic kidney disease Renal Failure 35 1089 1093 23883412 10.3109/0886022X.2013.817256

[ref12] Türkcü FM Şahin A Cingü AK Kaya S Yüksel H 2015 Serum omentin, resistin and tumour necrosis factor-alpha levels in Behcet patients with and without ocular involvement Graefe’s Archive for Clinical and Experimental Ophthalmology 253 1565 1568 10.1007/s00417-015-3016-0 25904298

[ref13] Yamawaki H Kuramoto J Kameshima S Usui T Okada M 2011 Omentin, a novel adipocytokine inhibits TNF-induced vascular inﬂammation in human endothelial cells Biochemical and Biophysical Research Communications 408 339 343 21514279 10.1016/j.bbrc.2011.04.039

[ref14] Urbanova M Dostalova I Trachta P Drapalova J Kavalkova P 2014 Serum concentrations and subcutaneous adipose tissue mRNA expression of omentin in morbid obesity and type 2 diabetes mellitus: the effect of very-low-calorie diet, physical activity and laparoscopic sleeve gastrectomy Physiological Research 63 207 218 24397804 10.33549/physiolres.932530

[ref15] Tan BK Adya R Farhatullah S Chen J Lehnert H 2010 Metformin treatment may increase omentin-1 levels in women with polycystic ovary syndrome Diabetes 59 3023 3031 20852028 10.2337/db10-0124 PMC2992762

[ref16] Luedde M Benz F Niedeggen J Vucur M Hippe HJ 2016 Elevated omentin serum levels predict long-term survival in critically ill patients Disease Markers 10 10.1155/2016/3149243 PMC5102724 27867249

[ref17] Loosen SH Koch A Tacke F Roderburg C Luedde T. 2019 The role of adipokines as circulating biomarkers in critical illness and sepsis International Journal of Molecular Sciences 20 4820 4820 10.3390/ijms20194820 PMC6801868 31569348

[ref18] Hillenbrand A Weiss M Knippschild U Wolf AM Huber-Lang M 2012 Sepsis-induced adipokine change with regard to ınsulin resistance International Journal of Inflammation 10 10.1155/2012/972368 PMC3261472 22272381

[ref19] Wang X Buechler NL Yoza BK McCall CE Vachharajani V 2016 Adiponectin treatment attenuates inflammatory response during early sepsis in obese mice Journal of Inflammation Research 9 167 174 27785087 10.2147/JIR.S119021 PMC5063563

[ref20] Karampela I Christodoulatos GS Dalamaga M. 2019 The role of adipose tissue and adipokines in sepsis: inflammatory and metabolic considerations, and the obesity paradox Current Obesity Reports 8 434 457 31637623 10.1007/s13679-019-00360-2

[ref21] Hillenbrand A Xu P Zhou S Blatz A Weiss M 2016 Circulating adipokine levels and prognostic value in septic patients International Journal of Inflammation 13 30 30 10.1186/s12950-016-0138-z PMC5012010 27601939

[ref22] Tzanela M Orfanos SE Tsirantonaki M Kotanidou A Sotiropoulou C 2006 Leptin alterations in the course of sepsis in humans In Vivo 20 565 570 16900791

[ref23] Koch A Sanson E Voigt S Helm A Trautwein C 2011 Serum adiponectin upon admission to the intensive care unit may predict mortality in critically ill patients Journal of Critical Care 26 166 174 20869198 10.1016/j.jcrc.2010.07.015

[ref24] Hillenbrand A Knippschild U Weiss M Schrezenmeier H Henne-Bruns D 2010 Sepsis induced changes of adipokines and cytokines - septic patients compared to morbidly obese patients BMC Surgery 9 10 10 10.1186/1471-2482-10-26 PMC2944119 20825686

[ref25] Pan HY Guo L Li Q 2010 Changes of serum omentin-1 levels in normal subjects and in patients with impaired glucose regulation and with newly diagnosed and untreated type 2 diabetes Diabetes Research and Clinical Practice 88 29 33 20129687 10.1016/j.diabres.2010.01.013

[ref26] Arjmand MH Moradi A Akbaric A Mehrad-Majd H. 2020 Clinical significance of circulating omentin levels in various malignant tumors: evidence from a systematic review and meta-analysis Cytokine 125 154869 154869 31585311 10.1016/j.cyto.2019.154869

[ref27] Petrov MS 2013 Nutrition, inflammation, and acute pancreatitis ISRN Inflammation 29 10.1155/2013/341410 PMC3893749 24490104

[ref28] Wu LM Sankaran SJ Plank LD Windsor JA Petrov MS 2014 Meta-analysis of gut barrier dysfunction in patients with acute pancreatitis British Journal of Surgery 101 1644 1656 10.1002/bjs.9665 25334028

[ref29] Kruis T Batra A Siegmund B. Bacterial 2013 – impact on the adipocyte compartment Frontiers in Immunology 4 510 510 10.3389/fimmu.2013.00510 PMC3881001 24432024

[ref30] Ferraria FL Bota DP Bross A Melot C Vincent JL 2001 Serial evaluation of the SOFA score to predict outcome in critically ill patients The Journal of the American Medical Association 286 1754 1758 11594901 10.1001/jama.286.14.1754

[ref31] Narumi T Watanabe T Kadowaki S Kinoshita D Yokoyama M 2014 Impact of serum omentin-1 levels on cardiac prognosis in patients with heart failure Cardiovascular Diabetology 13 84 84 24755035 10.1186/1475-2840-13-84 PMC4006671

[ref32] Tan YL Zheng XL Tang CK 2015 The protective functions of omentin in cardiovascular diseases Clinica Chimica Acta 448 98 106 10.1016/j.cca.2015.05.019 26079253

